# The Coronavirus E Protein: Assembly and Beyond

**DOI:** 10.3390/v4030363

**Published:** 2012-03-08

**Authors:** Travis R. Ruch, Carolyn E. Machamer

**Affiliations:** Department of Cell Biology, The Johns Hopkins University School of Medicine, 725 N. Wolfe St., Baltimore, MD 21205, USA; Email: truch2@jhmi.edu

**Keywords:** envelope protein, coronavirus assembly, ion channel, Golgi complex, membrane protein topology

## Abstract

The coronavirus E protein is a small membrane protein that has an important role in the assembly of virions. Recent studies have indicated that the E protein has functions during infection beyond assembly, including in virus egress and in the host stress response. Additionally, the E protein has ion channel activity, interacts with host proteins, and may have multiple membrane topologies. The goal of this review is to highlight the properties and functions of the E protein, and speculate on how they may be related.

## 1. Introduction

Coronaviruses (CoVs) are enveloped viruses with large positive-sense single-stranded RNA genomes. CoVs infect a variety of mammalian and avian species, and can cause serious disease in humans, as exemplified during the 2002–2003 outbreak of the severe acute respiratory syndrome (SARS).

The CoV E protein has a well-established role in the assembly of virions where it may induce membrane curvature or aid in membrane scission. Recent studies have expanded the role of CoV E beyond assembly. CoV E has ion channel activity *in vitro*. CoV E also is critical for the efficient trafficking of virions through the secretory pathway, a function that may be related to its ion channel activity. The CoV E protein has recently been shown to inhibit the host cell stress response, implicating it in pathogenesis. New interacting partners for E have been identified that expand the role of the protein during infection. How all of these potential properties of CoV E fit together to impact its function(s) is the focus of this review. 

## 2. Background

**Figure 1:**
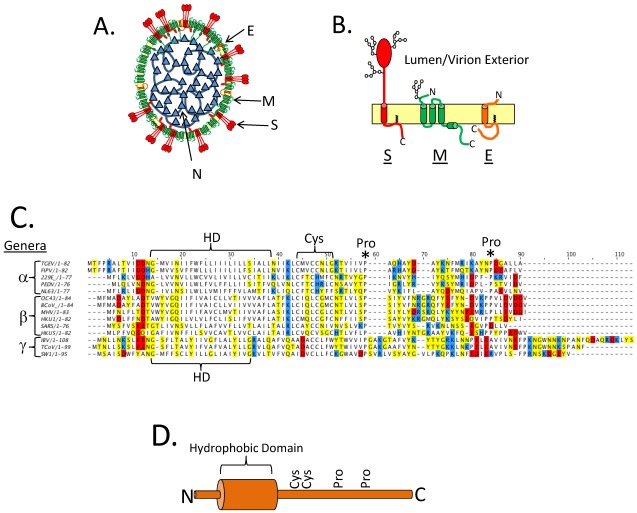
Primary structure of the Coronavirus (CoV) E protein. (**A**) A cartoon depicting a CoV virion. The structural proteins are labeled. (**B**) The three major CoV structural proteins in the virion envelope. Oligosaccharides are shown on S and M. A single topology is shown for E, see below for discussion on E protein topology. (**C**) A multiple sequence alignment of several different CoV E proteins. The hydrophobic domain (HD) is bracketed. The CoV genera (alpha, beta, and gamma) are denoted on the left of the multiple sequence alignment. Positively charged residues are shown in blue, negatively charged residues are shown in red, and polar uncharged residues are shown in yellow. The conserved Cys and Pro residues are labeled with a bracket or an asterisk, respectively. The multiple sequence alignment of CoV E proteins was carried out with ClusalW2 at the European Bioinformatics Institutes server, and Jalview software version 2 was used to generate the figure [16,17]. (**D**) Cartoon depiction of the E protein with the hydrophobic domain shown as a cylinder and the conserved Cys and Pro residues labeled.

The E protein, along with N, S, and M, are the major coronavirus structural proteins (Figure 1A,B). The N protein is a soluble protein and packages the RNA genome to form the nucleocapsid. The S protein has a single transmembrane domain, is found in the virion envelope, and serves as the attachment and fusion protein. The M protein has three transmembrane domains, is the most abundant protein in the virion envelope, and directs the assembly process through interactions with the other structural proteins (reviewed in [1,2]). CoV E is a small (76–109 amino acids) integral membrane protein, and has a single predicted hydrophobic domain (HD). It is usually encoded as the second or third message in a bi- or tri-cistronic mRNA [3,4]. The E protein is targeted to the Golgi region in infected cells and also when expressed from cDNA [5,6,7,8,9]. The membrane topology of CoV E is of considerable debate, having been reported as transmembrane or a membrane hairpin (see below) [5,10,11,12]. There are conserved membrane proximal cysteine residues that are targets for palmitoylation [7,13,14,15]. There are also conserved proline residues in the C-terminal tail (Figure 1C,D). Other than these similarities there is large variation in the primary sequence of the E proteins, which differ in size and sequence among virus groups often having <30% identity (Figure 1C). How or if these differences affect protein function is not known. 

## 3. CoV E and Virus Assembly

**Figure 2:**
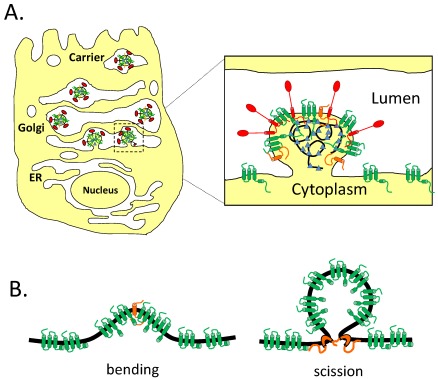
CoVs assemble and bud intracellularly at the ERGIC. (**A**) Newly formed virions bud into the lumen of the ERGIC and traverse the secretory pathway for egress. (**B**) Potential roles for E in assembly. The E protein is shown in orange and the M protein is shown in green. CoV E could help to bend membranes or play a role in membrane scission.

Unlike many other enveloped viruses, CoVs assemble and bud intracellularly at the ER-Golgi intermediate compartment (ERGIC) (Figure 2A) [18,19]. One of the early discoveries in CoV assembly was that formation of the virion envelope required only expression of M and E and not N. Originally observed for mouse hepatitis virus (MHV) [20], this property has been observed for infectious bronchitis virus (IBV) [5], transmissible gastroenteritis virus (TGEV) [21], and bovine coronavirus (BCoV) [21]. There has been considerable debate about the requirements for SARS-CoV envelope formation, with reports that M and N [22], M and E [23], and even M alone [24] can drive production of released vesicles. These results raise an interesting point about the efficacy of measuring virus-like particle (VLP) production in different cell types and expression systems. It is important to note that membrane proteins that form multimers can be secreted from cells in microvesicles [25]. Thus, overexpression of viral membrane proteins may lead to release in microvesicles, complicating the interpretation of VLP experiments. Additionally, the original reports for MHV, IBV, TGEV, and BCoV all used vaccinia-based expression systems. What has recently become apparent, at least for MHV [14], SARS CoV [26], and IBV [27], is that when using transient transfection to express the proteins from plasmids, the presence of N can greatly increase VLP yield. This result likely means that while not necessarily required for envelope formation, N plays an important role in forming a complete virion. This makes intuitive sense, and explains why empty virions are not readily purified from infected cells. However, the fact that M and E are sufficient for envelope formation is important information when considering the mechanism of assembly.

The ability of E and M to drive VLP formation clearly shows that E is important for assembly, but the mechanism is not well understood. The IBV E and M proteins interact via their cytoplasmic tails [28,29]. These interactions may be important for particle assembly [21]. Additionally, mutations introduced into the C-terminal tail of MHV E produced virions that were unstable, elongated, and may have resulted from failed scission events [30]. However, a version of IBV E with a heterologous HD was able to drive VLP formation and produce infectious virus, suggesting that the sequence of the HD was not important for assembly [29,31]. The E protein may also promote membrane rearrangements. MHV E can drive the intracellular formation of electron dense membranes derived from the ERGIC when expressed alone in BHK-21 cells [6]. Additionally, the HD of SARS-CoV E can drive the *in vitro* tubulation of dimyristoyl phosphatidylcholine (DMPC) membranes [32], although a version of the HD that was flanked with lysine residues did not have the same effect [33]. All of these observations point to a pivotal role for E in assembly, possibly in the scission of particles at the ERGIC, or in inducing membrane curvature (Figure 2B). Thus, it was surprising when versions of MHV, SARS-CoV, and TGEV lacking the E gene were shown to assemble virions, albeit to a lower degree than the corresponding wild-type viruses [34,35,36]. Furthermore, the E proteins from IBV, SARS-CoV, and BCoV could functionally replace MHV E in the context of infection, and TGEV E, which cannot substitute for MHV E, needed only a single amino acid change to complement MHVΔE [37]. This suggests that sequence specific protein-protein interactions between M and E are not required for assembly. One final piece of information came from the study of MHVΔE. Characterization of this virus showed that the production of infectious particles was severely compromised. However, after serial passage, revertants arose with a partial duplication of the M gene consisting of the N-terminus and all three transmembrane domains, but lacking most of the C-terminal cytoplasmic tail [38]. How a truncated version of M can functionally replace E is unknown, but it could allow for spacing between M proteins and disrupt lateral interactions with the M tails. Thus, the exact role of the E protein in assembly is not clear. It may be important for membrane curvature and/or scission directly, or it might alter the spacing of the M protein, which in turn is important for these effects. It is also worth noting the varied requirement for E in virion morphogenesis as revealed by the deletion studies in TGEV, MHV, and SARS-CoV [34,35,36,39,40]. The E protein is essential for TGEV production, but not necessary for MHV or SARS-CoV production. Why different viruses have a varied requirement for the E protein is not understood. One possibility is that a specific accessory protein could complement the assembly process in the absence of E for some CoVs.

## 4. Post-Translational Modifications

The best characterized post-translational modification (PTM) on E is the addition of palmitic acid onto membrane proximal cysteine residues. This modification has been reported for the E proteins of IBV [13], SARS-CoV [7], and MHV [14,15]. It is likely that palmitoylation alters the conformation of the tail in relation to the membrane. Palmitoylation is not important for proper targeting of the E protein [13,14,15]. The functional significance of palmitoylation has been well studied for MHV E, where it is important for assembly as judged both by VLP production and production of infectious virus [14,15]. These studies suggest that palmitoylation of E affects how it interacts with the M protein, possibly by allowing the E protein to gain access to specific lipid microdomains at the site of assembly [14].

Two other PTMs on the E protein have been reported, but not studied in depth. One study demonstrated that transiently expressed SARS-CoV E is N-glycosylated on asparagine 66 [10]; however the functional relevance of this is not known since the residue is on a portion of the protein reported to be in the cytoplasm (see discussion on topology below). Two studies have shown that SARS-CoV E can be ubiquitinated [41,42]. There is currently no known functional role for the ubiquitination of CoV E.

## 5. CoV E and the Cell Stress Response

There are varying reports on the role of the E protein in apoptosis. It was shown that overexpression of MHV E and epitope tagged SARS-CoV E induced apoptosis in some cultured cell lines [43,44]. However, a recent study compared the stress response of cells infected with SARS‑CoV to cells infected with SARS-CoVΔE. Using a microarray-based approach, it was shown that the virus lacking E induced a much more robust stress response than the wild-type virus. The virus lacking E also caused a greater degree of apoptosis compared to the wild-type virus [45]. This result, while contrary to the studies using overexpression, shows that SARS-CoV E may be anti-apoptotic during infection. The suppression of the host stress response by E may be important for down regulating the immune response and promoting pathogenesis. 

## 6. Protein-Protein Interactions

While the interaction between E and M has been established (reviewed in [2]), two recent studies have identified novel protein interactions with the SARS-CoV E protein. In the first study, cells were infected with SARS-CoV that had the E gene replaced with a C-terminally tagged version of E. Tandem affinity purification coupled with tandem mass spectrometry was carried out on the infected cells to find interacting proteins. Several candidates were identified, and the interaction of SARS-CoV E with the N-terminal ubiquitin-like domain-1 of SARS-CoV nsp3 protein was characterized. Nsp3 and CoV E colocalize during infection and nsp3 may be responsible for the ubiquitination of SARS‑CoV E [41]. Another report identified the tight junction protein PALS1 as an interactor with the C-terminal domain of SARS-CoV E [46]. The authors speculate that this interaction is important in the pathogenesis of SARS-CoV by promoting disassembly of tight junctions in lung epithelium after primary infection [46].

Understanding the protein-protein interactions of CoV E is an important step in elucidating the functions of the protein. Since the sequences of the E proteins are quite divergent, it will be interesting to determine if different CoV E proteins interact with the same host proteins, or are able to elicit a similar effect through interactions with other proteins.

## 7. The Ion Channel Activity of CoV E

Many viruses encode small proteins that have ion channel activity including Hepatitis C virus (HCV) p7 [47,48], influenza M2 [49], and picornavirus 2B [50]. All of these proteins are small (63–97 amino acids) and contain one or two transmembrane domains, making oligomerization a requirement for channel activity [51]. The role of these proteins varies between viruses, but all of them affect the secretory pathway. The M2 protein of influenza virus is the best characterized ion channel. M2 is a 97 amino acid type III membrane protein that forms a tetrameric pH activated proton channel [49]. The first described role for this activity was in the entry of the virus. Influenza virus is endocytosed after binding to a susceptible cell, and upon endosome acidification M2 facilitates the transfer of protons into the virion interior to aid in uncoating of the genome [52]. It was later appreciated that the M2 ion channel is active in infected cells, where M2 has an effect on the secretory pathway by raising the pH of the *trans*-Golgi network [53]. For some strains of influenza virus, this activity prevents the premature activation of the fusion protein [53,54,55].

HCV p7 is not as well studied as M2, but is relevant to the discussion because like CoVs, HCV assembles intracellularly and must navigate the host secretory pathway. HCV p7 is targeted to the ER and Golgi complex [56], where it is thought to oligomerize and act as an proton channel [48,57]. p7 is important for the assembly and release of HCV virions [58,59]. Recently, it was shown that p7 alkalinizes acidic organelles in the secretory pathway, and that M2 can complement a version of HCV lacking p7 [57]. How this activity promotes release of particles is not known. It may create an environment that protects virions from damage during exocytosis, or promote enlargement of Golgi cisternae to accommodate large cargo (see discussion of CoV E and release below). 

The channel activity of E was first demonstrated for SARS-CoV E in planar lipid bilayers, where it was found that a synthetic peptide corresponding to the protein could permeabilize bilayers to Na^+^ and K^+^, with a 10-fold preference for Na^+^ [60]. Further study generalized this property to other CoV groups, as IBV E, MHV E, and HCoV-229E E all have ion channel activity for monovalent cations [61]. Furthermore, the channels formed by MHV E and HCoV 229E E were inhibited by the broad spectrum Na^+^/H^+^ exchanger inhibitor hexamethylene amiloride (HMA) [61]. Since the E protein only has a single HD, formation of an ion channel would require oligomerization. This possibility was originally addressed using computational modeling, which predicted that the E protein HD could form stable dimers, trimers, and pentamers [62]. This prediction was further bolstered by several spectroscopic studies, which showed that the HD of SARS-CoV E forms a pentamer [63,64,65]. However, this result has not been confirmed for the other E proteins, or for the full length SARS-CoV E protein in a natural membrane. Nonetheless, predictions can be made from these structural data. For one, it appears that Asn15 in SARS-CoV E, and possibly the equivalent residue in other E proteins, is likely important either for oligomerization or ion conductance. Indeed, when alanine is substituted for this residue in a lysine-flanked peptide of the SARS-CoV E HD, conductance is largely inhibited [63]. Additionally, the equivalent residue in IBV E is required for perturbing the secretory pathway, suggesting a role for the ion channel in this effect [66]. 

The role of the CoV E ion channel in infection is not entirely clear, but studies using inhibitors offer some insight into its function. When HMA is added to the inoculum of either HCoV 229E or MHV, replication is inhibited. Adding more weight to this result, when HMA is added to the inoculum of MHVΔE no further growth defect is observed, showing that the inhibitory effect of HMA is dependent on the presence of the E protein [61]. While these studies suggest an important role for the ion channel activity of the E protein, it is not clear what step of viral replication is blocked by the drug. Two other studies have linked the putative ion channel activity with virus replication and release. A recombinant version of MHV carrying mutations in the HD was shown to have a defect in both assembly and release of particles [67]. A version of IBV carrying an a mutated version of E where the HD was replaced with that of an unrelated protein showed a defect in the release of infectious particles [31]. These results imply that the ion channel activity of E may be important in the release of virions from cells, and are discussed in the section below. 

## 8. Role of CoV E in Virion Release

### 8.1. The Mammalian Secretory Pathway and Large Cargo

**Figure 3:**
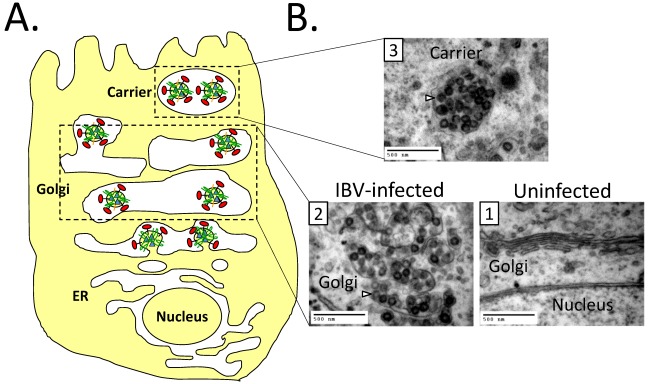
CoVs use the secretory pathway for egress. (**A**) A cartoon depicting virions within the Golgi complex. The Golgi cisternae are enlarged and fragmented during infection, possibly to aid in the trafficking of virions. (**B**) Transmission electron micrographs of uninfected or IBV infected Vero cells. (1) The Golgi complex of an uninfected cell; (2) The Golgi complex of an IBV infected cell with enlarged Golgi cisternae; (3) A putative virion carrier. Arrows denote virions. Scale bar is 500 nm. For images (2) and (3) Vero cells were infected at an moi of 0.1, and samples were fixed and processed 14 hrs post infection as described in [31].

Enveloped viruses that assemble intracellularly must navigate through the secretory pathway for release (Figure 3). Whether these viruses simply follow the canonical trafficking pathway, or modify it to handle the flux of large cargo is not known. To better understand this question, it is important to appreciate how the compartments used by CoVs are linked. The ERGIC is the intermediate compartment between the ER and Golgi. Its major function is to sort and concentrate cargo along the biosynthetic pathway. The ERGIC may mature as a unit to form the *cis*-Golgi, or it may be a more stable compartment [68]. From the ERGIC, cargo traffics to the Golgi complex. The Golgi complex is made up of polarized stacks of cisternae that are connected laterally into a ribbon. Golgi cisternae are generally between 700–1100 nm in diameter and <20 nm thick [69,70]. The polarity of the Golgi from *cis*-, *medial*-, to *trans*-Golgi is important for sequential processing and proper sorting of cargo. The mechanism by which cargo proteins move through the Golgi complex is controversial. It was originally thought this occurred by the maturation of cisternae [71]. This model was challenged when intra-Golgi transport was reconstituted *in vitro* [72,73]. However, some large cargo proteins such as procollagen I are too big to fit into normal transport vesicles. It was shown that procollagen I does not leave the Golgi cisternae during its transport, and instead the cisternae mature, as Golgi resident proteins are moved in a retrograde direction [74,75,76]. However, a different large cargo complex (reversible protein aggregates) is transported between the Golgi cisternae in “megavesicles” [77]. Thus, it is possible that both vesicle transport and cisternal maturation can move cargo through the Golgi complex in mammalian cells. 

Since virions are large cargo they would likely move through the Golgi complex via a cisternal maturation mechanism or in megavesicles. However, differences between trafficking of small and large cargo are not well understood [78]. Some insight into the trafficking of virions can be gained by examining how cells normally traffic very large cargo. Procollagen I forms 300 nm triple helical bundles in the ER, which move through the secretory pathway. As mentioned above, the procollagen filaments do not appear to move between the Golgi cisternae in vesicles, implying that they move through the stack via a cisternal maturation mechanism [74]. When procollagen bundles are present in the Golgi complex, the cisternae appear distended [79,80]. Additionally, the *trans*-Golgi network (TGN) expands in cells producing collagen, and this expansion is correlated with efficient release of procollagen [81]. The trafficking of lipoproteins induces similar changes in Golgi complex morphology. Chylomicrons are large (400 nm) complexes made up of apoB-48 bound to triacylglycerides [82]. These complexes are formed in the ER of intestinal epithelial cells and traffic through the Golgi complex during their biogenesis [83]. It has long been known that the induction of chylomicron production demands a substantial, but reversible, enlargement of the Golgi complex, as seen in electron micrographs of rat enterocytes after the animals were fed a fatty meal [84]. The changes induced in Golgi complex morphology for both procollagen and chylomicrons seem to underlie a necessary step in their trafficking. How the expansion of the Golgi complex aids in their secretion, and the mechanism used to induce the reversible change(s) are not known. However, it seems likely that enveloped viruses that assemble intracellularly may have evolved to exploit this mechanism. 

### 8.2. Impact of CoV Infection on the Secretory Pathway

Like the trafficking of large cargo, CoV infection leads to a rearrangement of endomembranes (Figure 3). It was initially observed that during MHV infection the Golgi complex is dispersed from its juxtanuclear position [85]. This result has recently been expanded upon using quantitative immune‑EM, where it was shown that several different types of membrane rearrangements occur during MHV infection. Most relevant to the discussion here, virions were seen in large carriers derived from Golgi/ERGIC membranes, suggesting that the rearrangement of the Golgi complex may be important for virion trafficking [86]. However, the mechanism underlying the change in Golgi complex morphology is not currently known. These changes appear similar to those observed for the large cargo described above, but may constitute a more dramatic change. 

Besides CoVs, several other enveloped viruses assemble intracellularly. If modifying the secretory pathway is a requirement for virion trafficking, one would predict that an alteration of secretory compartments would be a general feature of cells infected with these viruses. Indeed, many of them do modify the secretory pathway in striking ways. Flaviviruses assemble and bud into the ER [87]. Interestingly, during flavivirus infection Golgi proteins localize to the ER at the site of assembly during infection, but the function of this is not clear [88,89]. Bunyaviruses assemble by budding into the Golgi lumen [90,91]. During bunyavirus infection, the ERGIC and Golgi complex appear fragmented and vesiculated [92], but the underlying mechanism is not known. While it may not be surprising that viruses rearrange cellular membranes for their own use, it is puzzling that these viruses seemingly disrupt the very organelles that they depend on for egress. However, the apparent disruption may represent an exaggeration of the mechanisms used by the large cargo described above. It seems possible, or maybe even plausible, that these viruses are exploiting a pre-existing mechanism to facilitate the release of large cargo from the secretory pathway.

### 8.3. Effect of CoV E on the Secretory Pathway and Role in Release of Virions

The results discussed above suggest a connection between the morphological changes during CoV infection and virion trafficking. However, the relationship between the two has been elusive. Recent studies of CoV E have shed light on this issue, and it is now apparent that a link exists between the E protein, virion trafficking, and morphological changes in the Golgi complex. Several studies have shown that the E protein is important for virion release. The E protein of TGEV is essential for the propagation of the virus [39,40]. However, further characterization of TGEVΔE showed that virions were assembled in infected cells, but appeared arrested in the secretory pathway, apparently unable to traffic properly [36]. Analysis of a version of SARS-CoV lacking the E gene showed that virions accumulated intracellularly with aberrant material, suggesting that they may be sorted improperly in the absence of E [35]. As mentioned above, mutations introduced into the HD of MHV or IBV compromised the release of infectious particles from cells [31,67]. Additionally it was shown that expression of IBV E altered the secretory pathway in a manner dependent on a single residue within its HD, an effect that correlates with the release of particles [31,66]. These results have led to speculation that the E protein acts as an ion channel in the secretory pathway, driving the rearrangement of secretory organelles through the alteration of lumenal environments. This in turn leads to efficient trafficking of virions. This process may be analogous to what occurs for the large protein cargo mentioned above and should be further examined. 

## 9. Topology of CoV E

**Figure 4:**
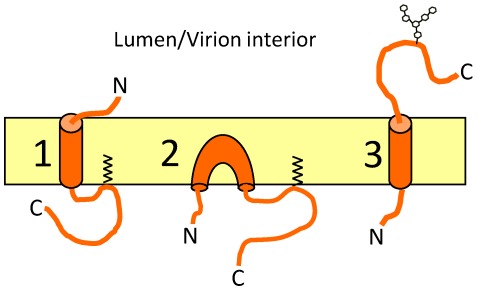
Topologies of the CoV E protein. The three proposed topologies of CoV E. (1) shows a type III membrane protein; (2) shows a membrane hairpin; and (3) shows a type II membrane protein with a putative N-linked oligosaccharide.

The topology of the CoV E protein is debated in the literature. The E protein does not have a canonical cleaved signal sequence [6], suggesting that the E protein is likely a type II or type III membrane protein. However, that is where the consensus ends. The C-terminus of TGEV E was detected at the surface of non-permeabilized infected cells, suggesting it adopted an N_cyto_C_exo_ topology (Figure 4(3)) [12]. IBV E has been reported as a transmembrane protein with the opposite topology, N_exo_C_cyto_ (Figure 4(1)) [5] The C-terminus of MHV E is present in the cytoplasm of infected cells, and on the inside of the virion [6]. Later it was shown, using an N-terminally FLAG tagged version of MHV E, that the N-terminus is present in the cytoplasm [11]. These results suggested an N_cyto_C_cyto_ topology, which is very strange for a protein with a single predicted HD, and suggests that the HD forms a membrane hairpin (Figure 4(2)). Multiple topologies have been reported for SARS-CoV E. Using transient expression of an N- or C-terminally FLAG tagged version of SARS-CoV E, both termini were present in the cytoplasm, supporting the membrane hairpin topology [10]. Additionally, this study also found that at least a portion of the expressed protein has its C-terminus modified with N-linked oligosaccharides, which would require the C-terminus to be in the ER lumen as a type II membrane protein [10]. More recently, an untagged version of SARS CoV E was shown to largely adopt an N_exo_C_cyto_ topology in infected cells and in cells transiently expressing the protein [9]. These results, along with those reported in [66] demonstrate the danger of using epitope tags to determine topology. Complicating matters, when prediction programs are used to model the topology of the E protein the results are not consistent with, and often are in direct opposition to, what has been observed experimentally (Table 1). 

**Table 1:** TM Pred [93], HMMTop [94], TMHMM 2.0 [95], MEMSAT3 [96], and MEMSAT-SVM [97] were used to predict the topology of four different CoV E proteins. The location of the N- and C-termini, as well as the number of predicted transmembrane passes are shown.

Program	IBV E			MHV E			SARS E			TGEV E		
	N	C	TMs	N	C	TMs	N	C	TMs	N	C	TMs
TM Pred	lumen	lumen	2	lumen	cyto	1	lumen	cyto	1	lumen	cyto	1
HMMTop	lumen	lumen	2	cyto	cyto	2	lumen	cyto	1	cyto	cyto	2
TMHMM 2.0	lumen	lumen	2	lumen	cyto	1	cyto	lumen	1	lumen	cyto	1
MEMSAT-SVM	lumen	lumen	2	lumen	lumen	2	lumen	lumen	2	cyto	lumen	1
MEMSAT3	cyto	cyto	2	lumen	cyto	1	lumen	lumen	2	lumen	cyto	1

It seems possible that the E protein may adopt multiple topologies during infection. Certainly the putative ion channel activity of E would require a transmembrane protein, but another function, such as membrane bending in assembly, could require a membrane hairpin. To address this possibility, versions of IBV E were developed that adopted either a transmembrane (by adding a canonical cleaved signal sequence) or a membrane hairpin (by putting a FLAG tag on the N-terminus). The results showed that only the transmembrane protein could alter the secretory pathway (possibly through ion channel activity), but was not as efficient at forming particles as judged by VLP production [66]. This may mean that a small portion of wild-type IBV E protein exists as a membrane hairpin and plays a role assembly. 

If CoV E is inserted into the membrane both in a transmembrane and membrane hairpin orientation, one question that arises is how a single protein might adopt multiple topologies. Certainly a type II or type III membrane protein could be generated using the signal recognition particle (SRP)-translocon pathway. However, can a membrane hairpin topology be achieved using SRP-translocon mediated membrane insertion? Caveolin is between 18–24 kD depending on the isoform, and has a ~32 amino acid long HD which is inserted as a hairpin [98]. The membrane insertion of caveolin is dependent on Sec61, suggesting that the ER translocon can generate proteins with a membrane hairpin topology [99]. Thus, it is possible that SRP-mediated membrane insertion could produce multiple topologies, including a membrane hairpin and a transmembrane protein. Nonetheless, the E protein is very different than caveolin, both in overall size and in the hydrophobicity of its HD. It is important to consider alternate membrane insertion pathways. Tail-anchored proteins are generally considered to have fewer than 30 amino acids following their transmembrane domain [100]. Many CoV E proteins are just outside of this range, but no studies have investigated the role of the GET pathway (guided entry of tail anchored proteins) in CoV E membrane insertion. Finally, the possibility exists that the E protein may be inserted into the membrane via a novel mechanism of post‑translational insertion [101]. It is interesting to speculate that CoV E may be inserted into the membrane via multiple mechanisms, resulting in distinct topologies.

One last point is that the topology of CoV E proteins may differ for different Co*Vs.* In addition to the variability shown in the prediction programs, there are also differences in the overall hydrophobicity and length of the transmembrane segments. How this impacts membrane topology is unknown, but it is worth noting that IBV E is the only E protein predicted to have two transmembrane domains. It is also possible that differences in the hydrophobic domains could affect the ion channel activity of the proteins. 

## 10. Perspectives on CoV E

The CoV E protein is an enigmatic protein. There is a high degree of variability in the behavior of the E proteins from different CoVs, including their requirement for assembly, virion trafficking, ion channel function, and method of expression in the genome. Yet, this protein is present in all known CoVs, suggesting it has a conserved role. It is interesting to speculate that the E protein from different CoVs has evolved to perform different functions. This could be due to cell-type specific requirements for each virus, or the ability of an accessory protein to complement a function normally carried out by E. Understanding how the E proteins from different CoVs vary is a crucial step in elucidating the mechanism of CoV assembly and egress. 

While the results reviewed here have better characterized the E protein, much work still remains to understand the mechanism of E protein function. The putative ion channel activity of E needs to be further examined. To this end, comprehensive electrophysiological analysis of the various E proteins in their natural setting (inserted into a Golgi membrane with normal post-translational modifications) needs to be carried out. It should also be noted that the E protein from different CoVs show variability in ion specificity, conductivity, and sensitivity to HMA. The reasons for this are not clear. Understanding how the ion channel activity of E affects replication is an important step in elucidating the function(s) of the protein with particular attention to cell types infected in the natural host. Recombinant viruses should be developed that encode other small ion channels along with E proteins that can support assembly but have no ion channel activity to determine if other channels can substitute for E. Additionally, analysis of the ion composition of the disrupted secretory pathway in IBV E expressing cells needs to be carried out to confirm that altered ion balance is responsible for the disruption.

The idea that the E protein alters the secretory pathway to allow for virion trafficking is intriguing. If this modification is required for efficient trafficking of virions due to their large size, one would predict that the E protein should facilitate the trafficking of other large cargo such as procollagen I and chylomicrons. To this end, trafficking assays should be developed which allow for the quantitative monitoring of procollagen I and chylomicrons through the Golgi complex in the presence or absence of the E protein. It is also possible that the morphological changes in the Golgi complex are the end result of microenvironment alterations required to protect virions from proteolytic damage during egress. To test this, the lumenal ion concentration of both cells expressing E and infected cells should be investigated using ratiometric imaging. 

The topology of E appears to be predominately N_exo_C_cyto_; however, the existence of mixed membrane topologies cannot be ruled out. One possibility is that a small portion of the E protein is inserted as a membrane hairpin, and that this conformation facilitates particle assembly, but not efficient trafficking of virions. To address this possibility, recombinant viruses expressing topologically constrained versions of E (like those developed in [66]) should be developed, and particle production as well as infectious particle release monitored. Additionally, *in vitro* membrane translocation assays should be carried out to determine the components necessary for membrane integration of the E protein (SRP mediated, GET mediated, or a novel route). 

Finally, because there is variability in the functions of E protein in different viruses, experiments to elucidate which functions are conserved and which are not should be performed. The ability of the different CoV E proteins to rescue the E deletions of other CoVs should be tested much the same way as they were for MHV [37]. In addition, membrane associated accessory proteins from different CoVs should be examined in this same assay to determine if any of them can substitute for E. Finally, the tandem-affinity approach used to identify interacting proteins for SARS-CoV [41] should be extended to other CoVs. 

It is now clear that the CoV E protein has multiple functions during infection, although they are not clearly understood. Also, the role(s) for the E protein in different CoVs may not be identical. A comprehensive model of E protein function will provide a better understanding of CoV biology and the mammalian secretory pathway, and may provide a target for therapeutic intervention during CoV infection. 

## Acknowledgments

We would like to thank Andy Pekosz for thoughtful discussion on the E protein. The work in the authors’ laboratory was funded by the NIH. TRR was supported by the Isaac Morris and Lucille Elizabeth Hay Graduate Fellowship. 

## Conflict of Interest

The authors declare no conflict of interest.
